# 

**Published:** 1966-04

**Authors:** 


### *Contents*

TUMOURS OF THE SKIN *D. C. Bodenham* 23

SEROLOGICAL TESTS FOR SYPHILIS *H. R. Cayton* 32

THE BIOCHEMICAL DETECTION OF INHERITED DISEASES *J. B. Holton* 35

NOTES AND NEWS . . . . . . . . . . . . 40

